# Genome-Wide Identification of the ABA Receptors Genes and Their Response to Abiotic Stress in Apple

**DOI:** 10.3390/plants9081028

**Published:** 2020-08-13

**Authors:** Hongmin Hou, Lingling Lv, Heqiang Huo, Hongyi Dai, Yugang Zhang

**Affiliations:** 1 College of Horticulture, Qingdao Agricultural University, Qingdao 266109, China; hmhou@qau.edu.cn (H.H.); 201301014@qau.edu.cn (L.L.); hydai@qau.edu.cn (H.D.); 2 Qingdao Key Laboratory of Genetic Development and Breeding in Horticultural Plants, Qingdao Agricultural University, Qingdao 266109, China; 3 Mid-Florida Research and Education Center, University of Florida, Apopka, FL 32703, USA; hhuo@ufl.edu

**Keywords:** *PYL* gene, abiotic stress, phylogenetic analysis, synteny analysis

## Abstract

The pyrabactin resistance (PYR)/PYR1-like (PYL)/regulatory components of ABA receptor (RCAR) (known as PYLs for short) have been identified and characterized as the ABA receptors in some plants. However, little is known about the details regarding *PYL* family genes in the apple (*Malus* *domestica*). In this study, we identified 13 apple *PYLs*, termed *MdPYL1-13*, which could be classified into four groups according to structural features of the amino acid sequence. The gene structures and conserved motifs analysis found that the majority of *MdPYL*s had a similar number of exons and similar conserved motif profile in the same group. In addition, 11 gene pairs were identified to exhibit synteny by synteny analysis between the apple and *Arabidopsis*. Furthermore, we investigated *MdPYL*s transcript level in various organs of the red-fleshed apple (*Malus* *sieversii* f. Neidzwetzkyana (Dieck) Langenf) ‘Xinjiang No.1’. The results suggested all *MdPYL*s within group I were expressed at relatively higher levels in all of the organs tested. However, the genes of group IV had little or no variation. Additionally, we found various hormone and stress-related cis-elements in the promoters of *MdPYLs* by analyzing cis-elements. Therefore, the expression levels of all *MdPYLs* were further detected under ABA, PEG, salt, and cold stresses in ‘Xinjiang No.1’ seedlings. We found that all *MdPYL*s except for *MdPYL11* were upregulated by ABA treatment, 10 genes were upregulated by PEG treatment, 12 genes were upregulated by NaCl treatment, and six genes were upregulated by cold treatment (4 °C) while seven genes were downregulated. Thus, these *MdPYLs* might be involved in the defense against abiotic stresses. In addition, the interaction between 13 MdPYLs and two 2C protein phosphatases in the apple (MdPP2C65 and MdPP2C72) was investigated in yeast two-hybrid assays. These results suggested that MdPYLs may bind to MdPP2C65 and MdPP2C72 in different manners and with different intensity. Our studies provide useful information for further investigating and researching the regulatory mechanisms of *PYL* family genes in response to abiotic stresses in the apple.

## 1. Introduction

Abscisic acid (ABA) is an important phytohormone involved in plant growth, plant development, and protection of plants against abiotic stresses [1,2]. The core components of ABA signaling included the regulatory component of ABA receptors (PYLs), group A type 2C protein phosphatases (PP2Cs), and the sucrose nonfermenting 1-related protein kinases (SnRKs) [3,4,5,6]. The PYLs interacted with PP2Cs to form PYL-PP2C heterodimer, which inhibited the phosphatase activity of key negative regulators PP2Cs, thereby leading to activation of pivotal positive regulators SnRK2s [7,8,9,10].

PYLs, as ABA receptors, belong to the StAR-related lipid-transfer (START) superfamily containing a ligand-binding pocket enclosed by four conserved loops, CL1-CL4 [4,6], which is important in ABA signal transduction pathway. To date, 14 PYLs containing the conserved structure have been recognized in the *Arabidopsis* genome [4,6,11], including *AtPYR1* (*Arabidopsis thaliana* pyrabactin resistance1) and 13 *AtPYLs* (*A. thaliana* PYR1-like1-13) [12]. Some *PYLs*, such as *AtPYR1*, *AtPYL1*, *AtPYL2*, and *AtPYL3* inhibited PP2Cs in an ABA-dependent manner, while others including *AtPYL4*, *AtPYL5*, *AtPYL6*, *AtPYL8*, *AtPYL9*, *AtPYL10*, and *AtPYL13* showed suppression of PP2Cs in the absence of ABA [10,12,13]. In addition, several of these genes were showed having diverse functions in the regulation of stomatal closure [14], drought resistance [5], leaf senescence [15], and root development [16,17,18]. 

Since the discovery of the *PYL* family in *Arabidopsis*, orthologous genes of *PYL* in other crops have been characterized at genome-wide levels, including six *PYLs* in sweet orange [19], eight *PYLs* in grape [20], 13 *PYLs* in rice [21], 14 *PYLs* in tomato [22], 14 *PYLs* in rubber tree [23], 24 *PYLs* in *Brassica rapa* [24], 27 *PYLs* in cotton [25], and 29 *PYLs* in tobacco [26]. Recently, the study regarding the roles of *PYLs* in other plant species besides *Arabidopsis* has begun to be reported, and the various function of the PYLs in plant development and stress resistance was emphasized. For instance, overexpression of rice *OsPYL3*, cotton *GhPYL10/12/26*, or maize *ZmPYL3/9/10/13* dramatically enhanced the sensitivity to ABA in transgenic *Arabidopsis* [27,28,29]. Similarly, overexpression of rice *OsPYL*/*RCAR5* or *OsPYL3/5/9/11* also increased the sensitivity to ABA and the tolerance to drought stress in transgenic rice [30,31,32]. Overexpressing Os*PYL9* significantly enhanced the tolerance to drought stress and leaf senescence induced by drought in transgenic *Arabidopsis* and rice plants [15]. Moreover, overexpression of *Arabidopsis AtPYL4* (A194T), *AtPYL5*, maize *ZmPYL8*/*9/12*, or cotton *GhPYL9-11A* greatly improved tolerance to drought in transgenic *Arabidopsis* plants, respectively [5,29,33,34]. In recent years, in addition to herbs, the function of the *PYLs* in woody and lianas plants has also been reported. Overexpression of *Populus trichocarpa PtPYRL1* or *PtPYRL5* in both *Arabidopsis* and poplar also enhanced ABA sensitivity and drought resistance [35]. In grape, *VvPYL1* could be combined with ABA and repress the ABI1 phosphatase activity [36]. The *VvPYL1* (*VvRCAR7*) exhibited high expression level in grape leaves treated by drought, salt, and cold stresses [37]. 

Unfortunately, in spite of an increase in reports in *Arabidopsis* and other plants such as maize, rice, and grape, the information concerning *PYLs* remains elusive in the apple. In this study, for the first time, we systematically identified the *MdPYLs* at the genome level in the *M. domestica* and performed their exon–intron structure and synteny analysis. Moreover, we investigated the spatiotemporal expression patterns of *MdPYLs* in various apple organs. Finally, we also examined expression patterns of *MdPYLs* under various abiotic stresses treatments. Our results will provide some clues for further investigating the function of *PYL* genes in apple tree growth and development, and resistance to abiotic stress. 

## 2. Results

### 2.1. Identification and Expansion Patterns of MdPYLs in the M. domestica Genome

**Figure 1:**
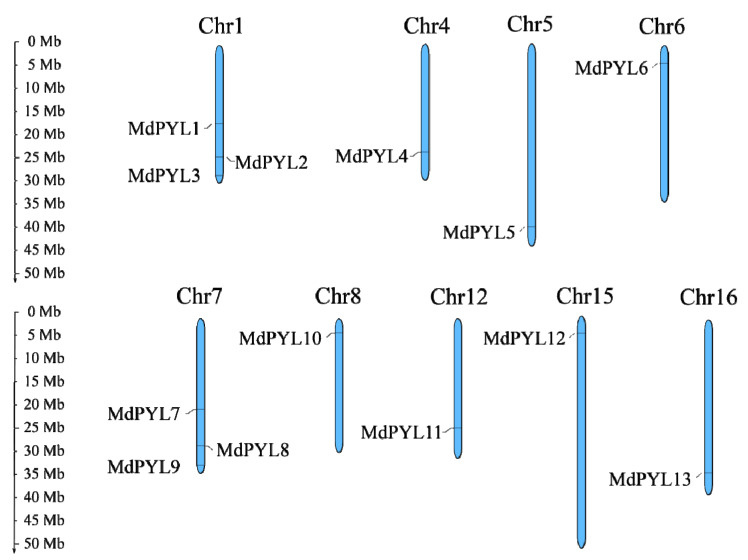
Chromosomal distribution and localization of *MdPYL*s. Only nine chromosomes contained *MdPYL*s are represented in this figure. The chromosome names are shown at the top of each chromosome. The chromosome scale is in millions of bases (Mb) on the left.

We determined that 13 putative *MdPYLs* were present in the *M. domestica* genome through BLASTP by using 14 AtPYL sequences as references. Then, we termed *MdPYL1* to *MdPYL13* based on their chromosomal order (Figure 1). Information regarding *MdPYL*s was provided in Table 1 consisting of the gene name, gene ID, length of coding DNA sequence (CDS), theoretical isoelectric point (pI), molecular weight (MW), and length of protein. The sequence analysis revealed that the *MdPYL*s lengths changed obviously in size, ranging from 558 bp (*MdPYL5*) to 3252 bp (*MdPYL1*), with one to four exons in each sequence. However, the lengths of CDS and the corresponding MdPYL proteins both had no significant difference, varying from 555 bp (*MdPYL1* and *MdPYL7*) to 777 bp (*MdPYL4*) and from 184 aa (MdPYL1 and MdPYL7) to 258 aa (MdPYL4), respectively. The predicted MW and pI values of these proteins ranged respectively from 20.13 kDa (MdPYL5) to 27.66 kDa (MdPYL4) and 5.02 (MdPYL10) to 8.30 (MdPYL11) (Table 1).

Moreover, according to available annotation information of the GDR database, 13 putative *MdPYL*s were distributed on nine chromosomes including chromosome 1, 4, 5, 6, 7, 8, 12, 15, and 16 (Figure 1). Three *MdPYL*s were present on chromosome 1 and 7, respectively, and one *MdPYL* on the remaining chromosomes (Figure 1).

### 2.2. Phylogenetic Analysis of the MdPYLs Family

**Figure 2:**
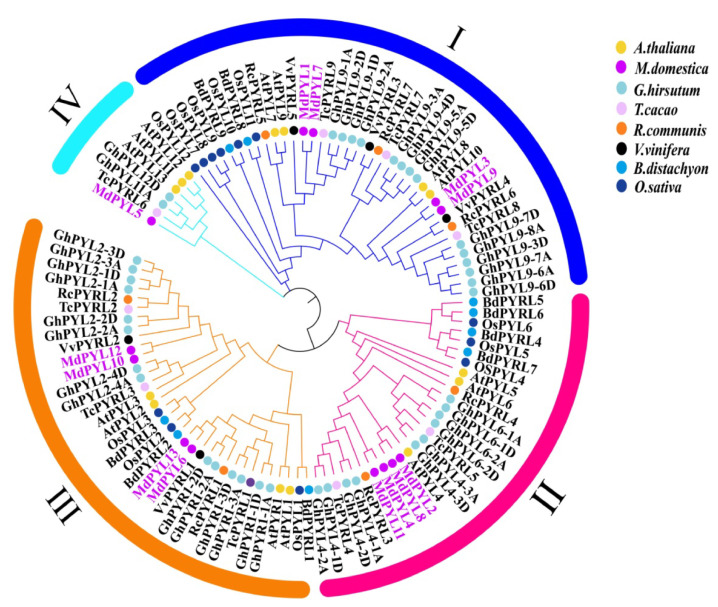
Phylogenetic analysis of *PYLs* from seven species. Different groups of *MdPYLs* are indicated in different colors bars. Circles of different colors denote *PYLs* from different species, including 14 *AtPYLs* from *A. thaliana* (yellow dot), 13 *MdPYLs* from *M. domestica* (purple dot), 7 *RcPYLs* from *R. communis* (orange dot), 5 *VvPYLs* from *V. vinifera* (black dot), 9 *BdPYLs* from *B. distachyon* (blue dot), 12 *OsPYLs* from *O. sativa* (navy blue dot), 9 *TcPYLs* from *T. cacao* (pink dot) and 40 *GhPYLs* from *G. hirsutum* (light blue dot). All amino acid sequences used in this analysis are listed in Supplementary Table S1.

We constructed a phylogenetic tree based on 109 protein sequences of *PYL*s from seven species, including 14 *AtPYLs* from *A. thaliana*, 13 *MdPYLs* from *M. domestica*, 7 *RcPYLs* from *R. communis*, 5 *VvPYLs* from *V. vinifera*, 9 *BdPYLs* from *B. distachyon*, 12 *OsPYLs* from *O. sativa*, 9 *TcPYLs* from *T. cacao*, and 40 *GhPYLs* from *G. hirsutum* (Table S1) via MEGA7 software using the neighbor-joining algorithm. In general, the 109 plant *PYL*s tested were classified into four subgroups and named as group I–IV (Figure 2, Tables S1 and S2). However, the plant PYL family evolutionary relationship was diverse in various species of plants. As expected, *MdPYLs* from apple generally showed closer genetic relationships to *PYLs* of dicotyledonous angiosperms (*V. vinifera*, *A. thaliana*, *G. hirsutum*, *R. communi*, and *T. cacao*) than monocotyledonous angiosperms (*O. sativa* and *B. distachyon*) (Figure 2). The results suggested plant *PYLs* classifying into the same groups might have similar functions.

### 2.3. Gene Structure and Duplication Analysis of MdPYLs

**Figure 3:**
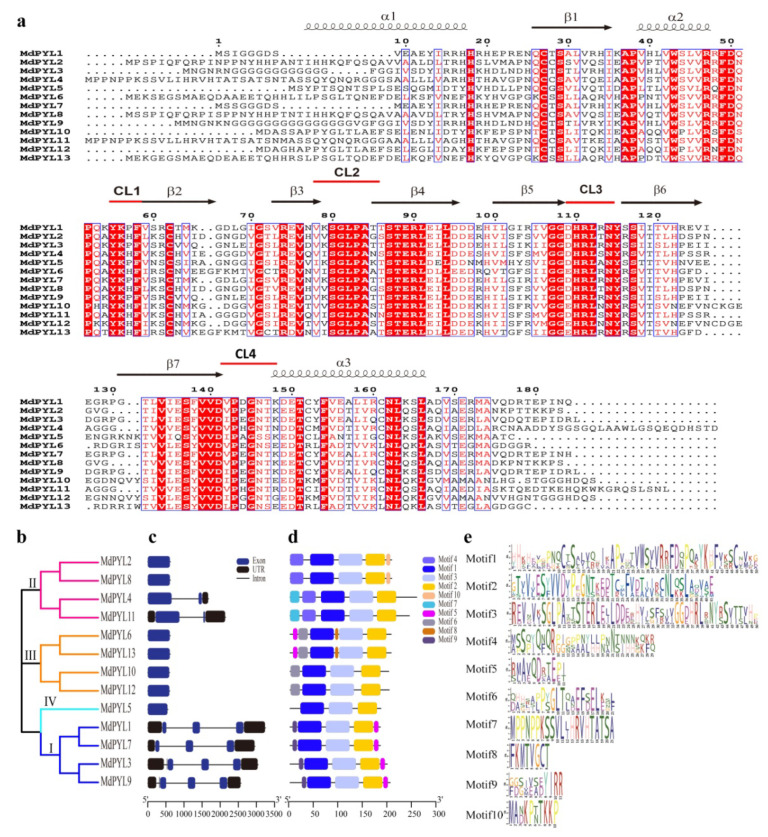
Amino acid sequence alignment, phylogenetic relationships, gene structure, and conserved motifs of *MdPYLs*. (**a**) Amino acid sequence alignment and secondary structural elements of the 13 MdPYLs. CL1–CL4 shown with red lines represent four conserved ABA receptor regions. (**b**) The phylogenetic tree of *MdPYLs*. Bars of different colors represent different groups of *PYLs*. (**c**) Exon–intron structure of apple *MdPYL*s. Black boxes denote untranslated regions; blue boxes denote exons; black lines denote introns. (**d**) Distributions of ten conserved motifs in *MdPYL*s. (**e**) The sequence logos of ten conserved motifs.

**Figure 4:**
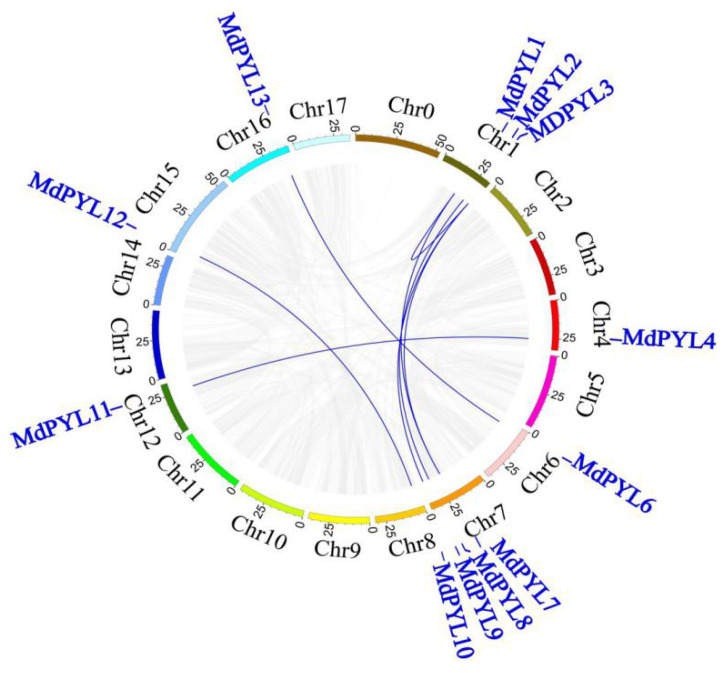
The synteny analysis of *MdPYL*s in the apple. Gray lines denote all synteny blocks in the apple genome, and the blue lines denote *MdPYL* duplicated gene pairs.

To study the structure and expansion mechanisms of *MdPYLs* gene family, we carried out the analysis of amino acid alignment, phylogenetic tree, exon–intron structures, the conserved motifs, and segmental duplications (Figure 3). In general, 13 MdPYLs protein all contained three α-helixes (α1–α4), seven-stranded β-sheet (β1–β7), and four highly conserved surface loops (CL1–CL4). (Figure 3a). The structure had been well characterized in the *AtPYLs* gene family, which involved in ABA binding and inhibition of PP2Cs [38]. Moreover, the majority of *MdPYL*s had a similar profile of exons, and length of ORFs in the same group (Figure 3b,c). For example, all *MdPYL*s within group I included three exons, while *MdPYL*s in groups II and III had only one exon (no intron), except *MdPYL4* and *MdPYL11* both contained two exons (Figure 3b,c). Meanwhile, all of *MdPYL*s shared highly conserved motif 1, 2, and 3, and *MdPYL*s belonging to the same group seemed to have a similar conserved motif distribution. For example, in addition to the motif 1, 2, and 3, all of group I, II, and III respectively shared the highly conserved motifs 7 and 10, motif 1, and motif 8 (Figure 3d,e). To further understand the expansion mechanism of the *MdPYL*s, we also examined segmental and tandem duplications within the apple genome. No tandem duplications were identified and eight segmental duplication were found in 12 pairs of duplicated genomic regions, respectively, including (*MdPYL1/MdPYL3*, *MdPYL1/7*, *MdPYL2/MdPYL8*, *MdPYL3/MdPYL7*, *MdPYL3/MdPYL9*, *MdPYL4/ MdPYL11*, *MdPYL6/MdPYL13*, *MdPYL10/MdPYL12*) (Figure 4).

### 2.4. Evolutionary Relationship of PYLs Between Apple and Arabidopsis

**Figure 5:**
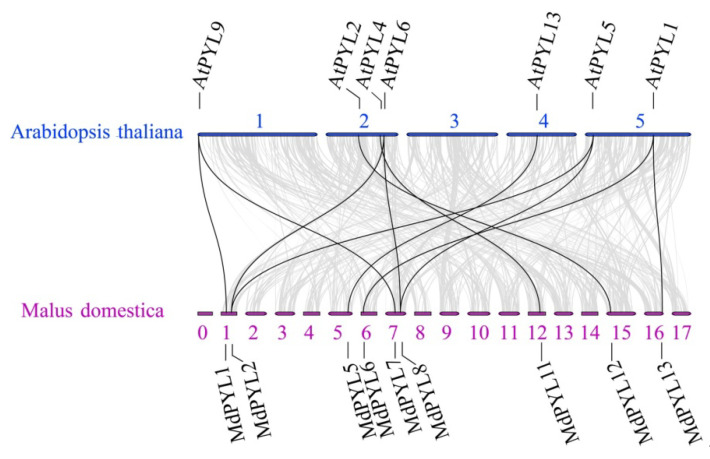
Synteny analysis of *PYL*s between apple and *A. thaliana*. Gray lines denote the collinear blocks between apple and *A. thaliana* genomes and the black lines denote the syntenic gene pairs of *PYLs*. Blue and purple lines represent respectively the apple chromosomes (0–17) and *Arabidopsis* chromosomes (1–5).

**Figure 6:**
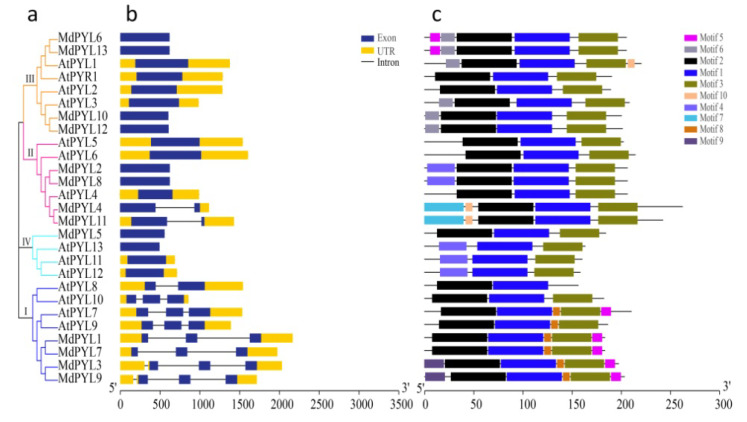
Phylogenetic relationships, gene structure, and conserved motifs of *MdPYLs* and *AtPYLs*. (**a**). The phylogenetic tree of *PYLs* from the apple and *Arabidopsis*. Bars of different colors represent different groups of *PYLs.* (**b**). Exon–intron structure of *MdPYL*s and *AtPYLs*. Yellow boxes denote untranslated regions; blue boxes denote exons; black lines denote introns. (**c**). Distributions of ten conserved motifs in *MdPYL*s and *AtPYLs*.

Comparative genomic analysis between the apple and *Arabidopsis* genomes found the syntenies were clear and included the following ortholog pairs: *MdPYL1*, *7*-*AtPYL9*; *MdPYL2*, *8*-*AtPYL5*, *6*; *MdPYL5*-*AtPYL13*; *MdPYL6*, *13*-*AtPYL1*; *MdPYL11*-*AtPYL4* and *MdPYL12*-*AtPYL2* (Figure 5). Furthermore, *AtPYLs* and *MdPYLs* from the same group had similar exon–intron structures (Figure 6a,b). Meanwhile, all of the *AtPYLs* and *MdPYLs* shared highly conserved motifs 1, 2, and 3, except *AtPYL11*, *12*, and *13* (Figure 6c). Significantly, many ortholog pairs between the apple and *Arabidopsis* genomes included nearly the same structure and conserved motif. For example, in *MdPYL6*, *13*-*AtPYL1* gene pairs belonging to group III all had only one exon and conserved motif 6. *MdPYL1*,*7*-*AtPYL9* gene pairs belonging to group I all had three exons and conserved motif 8 (Figure 5,6). The result will provide further insight into the functions of apple *MdPYL*s.

### 2.5. Cis-Element Analysis of the MdPYLs Promoter in Apple

**Figure 7:**
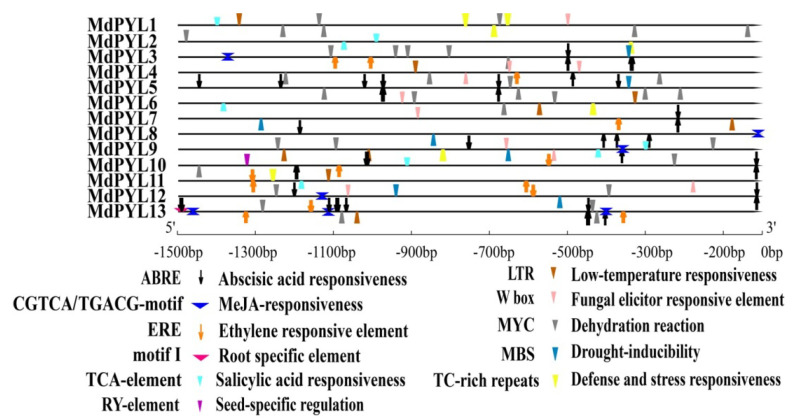
The cis-elements analysis in *MdPYL* promoters. The 1500 bp promoter sequences of 13 *MdPYLs* were analyzed by PlantCARE. The up and down directions respectively denote the cis-elements existing in the plus or minus strand of *MdPYL* promoters.

Previous works have indicated that many *MdPYLs* involved in the response to different abiotic stresses. To further forecast the potential functions of *MdPYLs*, we analyzed the cis-acting elements involved in abiotic stresses responses in the *MdPYLs* promoter regions using PlantCARE software (Table S3,S4). In total, three abiotic stress-related and four hormone-related elements were discovered in the promoter of 13 *MdPYLs*, including drought-inducibility elements (MBS), defense and stress-responsive elements (TC-rich repeats), dehydration reaction elements (MYC), low-temperature-responsive elements (LTR), abscisic acid-responsive elements (ABRE), salicylic acid-responsive elements (TCA-element), ethylene-responsive elements (ERE), and MeJA-responsive elements (CGTCA-motif, TGACG-motif) (Figure 7). All *MdPYLs* promoter regions contained at least three cis-elements (Figure 7). For example, the *MdPYL7* promoter had seven stress-responsive elements including ABRE, MBS, MYC, LTR, TC-rich repeats, CGTCA, and TCA-element (Figure 7). The *MdPYL13* promoter region contained six stress-responsive elements including ABRE, MBS, MYC, LTR, ERE, and CGTCA-element. Moreover, the *MdPYL2*, *5*, *8* promoter included three, the *MdPYL1*, *6*, *7*, *10*, *11* promoter comprised four, and the *MdPYL3*, *4*, *12* promoter contained five stress-responsive elements. Furthermore, the *MdPYLs* promoter had two tissue-specifc cis-elements (root-specific regulatory element and seed-specific regulation element) (Figure 7). The results concluded that the expression of *MdPYLs* with different cis-elements may differ in response to various abiotic stresses.

### 2.6. Expression Profiles of Apple MdPYLs in Diverse Organs

**Figure 8:**
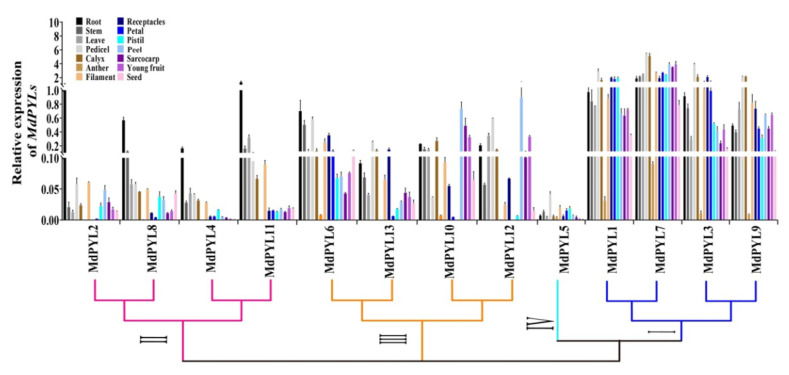
Tissue-specific expression of *MdPYLs*. Quantitative RT-PCR was performed on roots, stems, leaves, pedicels, calyxes, anthers, filaments, receptacles, petals, pistils, peels, sarcocarps, young fruits, and seeds. The expression of *MdPYL2* in roots was set to 1 and *MdActin* was used as an internal control. The relative expression levels of genes were calculated based on the 2-ΔΔCt method, and values are the mean ± SD obtained from three biological replicates.

To further understand the expression profiles of *MdPYL*s in apple development, we detected their expression levels in different tissues and organs from Chinese wild apple clone ‘Xinjiang No.1’ using qRT-PCR. In general, most *MdPYLs* were expressed differently in various organs, showing they might have different functions (Figure 8). Notably, *MdPYL5 (*group IV) had little or almost no expression in the organs tested. In contrast, the expression levels of all *MdPYLs* within group I, including *MdPYL1*, *MdPYL3*, *MdPYL7*, and *MdPYL9*, were higher in all of the organs tested, and were more than 20 times of that in group IV. Furthermore, *MdPYLs* of group II, including *MdPYL2*, *MdPYL4*, *MdPYL8*, and *MdPYL11*, were preferentially expressed in the root and their expression level were more than fivefold of the other organs. At the same time, we found *MdPYLs* within group III expression exhibited the relatively higher levels than *MdPYLs* of group II in all tissue analyzed except for root. For example, the expression of *MdPYL10* and *MdPYL12* in peel, sarcocarp, and young fruit were over fivefold of all genes within group II (Figure 8). These results suggested *MdPYLs* might have a distinct effect on growth and development of the apple tree.

### 2.7. Expression Profiles of MdPYLs in Response to Various Abiotic Stresses

**Figure 9:**
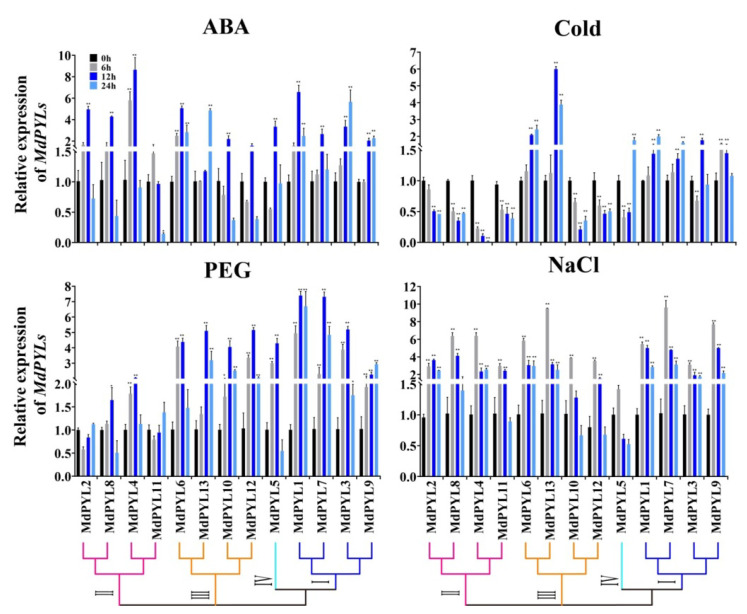
Expression of the apple *MdPYLs* under ABA, PEG, cold (4 °C), and salt treatment in the tissue-cultured apple seedlings (Xinjiang No.1). The gene relative expression was calculated using the 2-ΔΔCt method with *MdActin* as an internal control, and value represents mean ± SD of three biological replicates. Asterisks indicated values that are significantly different from CK (0 h) (* *p* < 0.05, ** *p* < 0.01, one-way ANOVA).

PYLs, as ABA receptors, might play a vital function in abiotic stress signaling pathways. Therefore, we detected the expression of all 13 apple *MdPYLs* in Chinese wild apple clone ‘Xinjiang No.1’ tissue-cultured seedlings upon various abiotic stress treatments (including ABA, NaCl, PEG, cold) by carrying out qRT-PCR. Genes with expression levels that were increased or decreased more than two-fold, were used for the following analyses (Figure 9).

In our study, we found that the expression of almost all of *MdPYL*s were significantly changed by ABA, PEG, NaCl, and cold. For example, the expression of all *MdPYL*s except for *MdPYL11* exhibited significant increases by ABA treatment (Figure 9). Nine genes (*MdPYL1*, *MdPYL2*, *MdPYL4*, *MdPYL5*, *MdPYL6*, *MdPYL7*, *MdPYL8*, *MdPYL10*, and *MdPYL12*) reached the peak at 12 h after ABA treatment, while three genes (*MdPYL3*, *MdPYL9*, and *MdPYL13*) reached the peak at 24 h. Notably, *MdPYL1*, *MdPYL2*, *MdPYL3*, *MdPYL4*, *MdPYL6*, *MdPYL8*, and *MdPYL13* all were upregulated more than fourfold after 12 h or 24 treatment, respectively. Similarly, following with PEG treatment, 10 genes (*MdPYL1*, *MdPYL2*, *MdPYL3*, *MdPYL5*, *MdPYL6*, *MdPYL7*, *MdPYL9*, *MdPYL10*, *MdPYL12*, and *MdPYL13*) were sharply upregulated in expression after 6 h of treatment and reached the maximum at 12 h, while the expression of remaining three apple *MdPYL*s had no obvious changes (Figure 9). The expression of all *MdPYLs* within group I, group III, and group IV were over fourfold higher than CK (0 h) at 12 h after PEG treatment. After NaCl treatment, the expression of all *MdPYL*s except for *MdPYL5* showed significant increases and surged to the peak at 6 h (more than threefold of CK) (Figure 9). Following cold treatment (4 °C), all *MdPYLs* within group I, and *MdPYL6*, *13* within group III demonstrated increased expression. More remarkably, *MdPYL*13 was upregulated more than sixfold after 6 h treatment. In contrast, all *MdPYLs* within group II, and *MdPYL10*, *12* within group III were significantly downregulated (Figure 9). 

### 2.8. Interaction between MdPYL and MdPP2C Proteins

**Figure 10:**
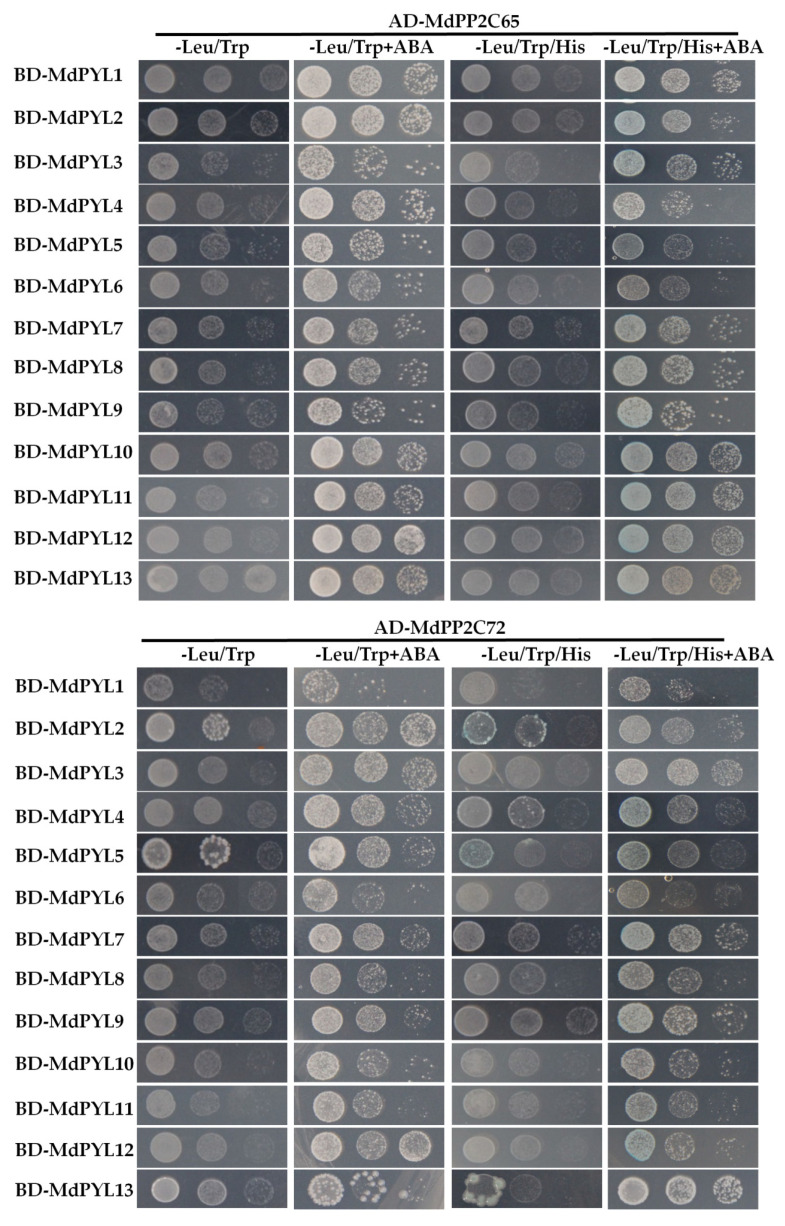
Interactions between MdPYLs and MdPP2Cs in yeast. Co-transformed positive yeast cells dilutions (10^−1^, 10^−2^, and 10^−3^) were spotted onto the plates. Interaction was determined by growth assay on SD-Leu/-Trp/-His/X-α-Gal/3-AT medium in the presence and absence of 10 μM ABA.

PYLs can interact with PP2Cs to form PYL-ABA-PP2C triple complexes promoting the ABA signals transmission [1]. However, other studies suggested that PYLs can bind PP2Cs in an ABA-dependent or ABA-independent manner in yeast two-hybrid (Y2H) assays [4,12]. In this study, 13 MdPYLs and 2 MdPP2Cs were isolated (Figure S1) to detect their potential interactions in the yeast two-hybrid system. The full coding sequence of all MdPYLs were respectively fused to the DNA-binding domain (BD) of pGBKT7 vector, while two MdPP2Cs were respectively fused with activation domain (AD) of pGADT7 vector. After co-transformed into the Y2H Gold yeast strain, all of yeast cells were capable of growth on SD-Leu/-Trp medium in the absence or presence of ABA. However, the co-transformed yeast cells grew faster in medium with ABA (Figure 10). Interaction between 13 MdPYLs and two MdPP2Cs was determined by growth assay on SD-Leu/-Trp/-His/X-α-Gal/3-AT medium with and without 10 μM ABA. In this experiment, all MdPYLs interacted with MdPP2C65 in an ABA-dependent manner, and these interactions were considerably enhanced in presence of ABA. However, MdPYLs differentially interacted with MdPP2C72. MdPYL1, MdPYL3, MdPYL6, MdPYL10, MdPYL11, and MdPYL12 interacted with MdPP2C72 in an ABA-dependent manner. MdPYL4, MdPYL7, MdPYL8, and MdPYL9 showed weak interaction signals with MdPP2C72 in the absence of ABA. Furthermore, we found that MdPYL2,5,13 showed stronger interaction signals activating the reporter gene X-α-Gal with MdPP2C72 in an ABA-independent manner (Figure 10). In addition, all MdPYLs and MdPP2Cs had no obvious self-activation (Figure S2). These results suggested that MdPYLs may bind to MdPP2C65 and MdPP2C72 in different manners and with different affinities.

## 3. Discussion

### 3.1. Gene Functional Diversification of Apple MdPYLs

*PYL*s encoding an ABA receptor family contained a similar ligand-binding pocket embraced by four conserved surface loops (CL1–CL4). In the study, 13 *MdPYLs* shared the typical structure were identified in the apple, with an almost equal number of *AtPYLs* from *Arabidopsis* (Figure 2 and Figure 3a). Comparative genomic analysis found that nine *MdPYLs* (*MdPYL1*, *2*, *5*, *6*, *7*, *8*, *11*, *12*, *13*) were positioned in syntenic regions of the apple and *Arabidopsis* genomes (Figure 5), and the other four *MdPYLs* (*MdPYL3*, *4*, *6*, *9*) were formed by segment duplication events in the apple genome (Figure 4). Meanwhile, almost all of the ortholog pairs between apple and *Arabidopsis* genomes also contained similar exon–intron structures and conserved motifs. The results suggested that the function of *PYLs* gene might be relatively conserved between the apple and *Arabidopsis*. To date, the functions of many *AtPYL*s have been reported in *Arabidopsis* [15,16,17,18,22,39]. According to *MdPYLs* expression data (Figure 8 and Figure 9) and the information known concerning their *Arabidopsis* counterparts, we will better predict the probable functions of apple *MdPYL*s.

### 3.2. Expression Profiles of Apple PYLs and Their Potential Functions in Various Apple Organs 

ABA is an important phytohormone for plant growth and development. PYL family members, as ABA receptors, have been identified and characterized in many plants including *Arabidopsis*, maize, rice, grape, rubber tree, and so on. To date, their expression profiles in various tissues have been revealed substantial differences among *PYL* genes for many plants. For example, some *PYLs* respectively displayed relatively higher levels in seeds of soybean, the callus of *B. rapa*, and the latex of rubber tree than other tissues [23,24,40,41]. Moreover, most *OsPYLs* were expressed in all organs of rice, *OsPYL3* and *OsPYL5* were predominantly expressed in leaves, and *OsPYL1* in roots [42]. Similarly, in maize, *ZmPYL11* was upregulated in leaves and *ZmPYL6* and *ZmPYL10* in roots [43]. In tomato, *PYR/PYL/RCAR* genes (10g085310 and 3g095780) had high levels of expression in root, and 1g095700 showed predominant expression in leaf, 12g055990 and 8g082180 in fruit tissues [22]. In grape, most of the *VyPYLs* genes, especially *VyPYL1*, *VyPYL4a*, and *VyPYL7*-*9* were expressed relatively strongly in roots, stems, and tendrils [44]. These reports indicated that *PYLs* play an important role in plant growth and development. The function of PYLs in *Arabidopsis* has been studied to support this idea. For example, overexpression of *RCAR11*, *RCAR12*, *RCAR13*, or *RCAR14* inhibited the germination and root growth of transgenic *Arabidopsis* in an ABA-treated condition [45]. *AtPYL9* promoted ABA-induced leaf senescence in transgenic *Arabidopsis* [15]. *AtPYL8* promoted lateral root growth by interacting with AtMYB77 and enhancing its transcriptional activity [18]. However, *MdPYLs* expression patterns and functions are still unclear in apple. Therefore, we speculated the roles of the apple *MdPYLs* based on qRT-PCR results in different tissues, and the information known of *Arabidopsis* counterparts (Figure 5) and homology genes of other species (Figure 2).

In general, the apple *MdPYLs* exhibited qualitatively and quantitatively distinct expression profiles. However, *MdPYLs* clustered to the same group, especially the eight pairs of genes in duplicated genomic regions, showed similar expression patterns, respectively (Figure 8), suggesting that they might have similar functions. For example, all *MdPYL*s within group I, including *MdPYL1*, *MdPYL3*, *MdPYL7*, and *MdPYL9*, exhibited relatively higher expression levels in all of the tissues tested compared to *PYLs* in other groups (Figure 8). Thus, we speculated that *MdPYLs* of the group I might function in the regulation of plant different growing and developmental stages. The function of homologous genes in other species has been studied to support this hypothesis. For instance, *AtPYL8* and *AtPYL9*, which are orthologs of *MdPYL1*, *7*, are involved in controlling root sensitivity to ABA [16]. *OsPYL8* and *OsPYL9*, belonging to group I, were specifically expressed in the rice endosperms and positively regulated by ABA during seed germination [46]. Furthermore, *MdPYL*s of group II, including *MdPYL2*, *MdPYL4*, *MdPYL8*, and *MdPYL11* were preferentially expressed in the root (Figure 8), indicating that these genes might play a role in root development. Indeed, the *AtPYL5*/*AtPYL6* gene, as the ortholog of *MdPYL2*/*MdPYL8*, was expressed at the higher levels in root and might play a critical role for the regulation of root growth and root system architecture [5]. In short, these results indicated the diverse biological functions of different *PYLs* in plant growth and development.

### 3.3. Apple MdPYLs Were Responsive to Various Abiotic Stresses

To date, *PYL*s, as important ABA receptors, have been found to play crucial roles in responding to various abiotic stresses in plants. Overexpressing *pRD29A::PYL9* dramatically promoted drought resistance and leaf senescence induced by drought in *Arabidopsis* and rice plants [15]. Similarly, overexpression of *AtPYL4*, *5*, *7*, *8*, and *13* also respectively enhanced drought tolerance and showed hypersensitivity to ABA during early seedling development in transgenic *Arabidopsis* [47]. Furthermore, overexpressing ABA receptors (*RCAR11*-*14)* also increased drought resistance and accelerated stress-responsive gene expression in transgenic *Arabidopsis* [45]. With the deepening study in *Arabidopsis*, the functions of orthologous PYL proteins’ responses to stresses were reported in several plants, such as rice, cotton, tomato, and grape. In rice, ectopic expression of *OsPYL3* improved cold and drought tolerance in transgenic *Arabidopsis* [31]. Constitutive expression of *OsPYL/RCAR5* increased drought and salt stress tolerance in rice [30]. In *Gossypium*, the expression level of many *GhPYLs* were downregulated by ABA treatment and upregulated by osmotic stress [27,34]. Overexpression of *GhPYL9-11A*, *GhPYL10*, *GhPYL12*, and *GhPYL26* improved the tolerance to drought stress in transgenic *Arabidopsis* [27,34]. Heterologous expression of tomato *ABA receptors* (6g050500 or 3g007310, or 8g076960) and *Vitis yeshanensis VyPYL9* all enhanced transgenic *Arabidopsis* drought resistance [22,44]. However, there is only a handful information concerning these genes response to stress in apple. Thus, we investigated the response of all 13 *MdPYLs* to various abiotic stress conditions in the study (Figure 9), and found that 12 *MdPYLs* (except *MdPYL11*), 12 *MdPYLs* (except *MdPYL5*), and 10 *MdPYLs* (except *MdPYL4*, *MdPYL8*, and *MdPYL11*) had significant expression changes when treated with ABA, salinity, and PEG, respectively (Figure 9). The results were consistent with the function of PYLs in *Arabidopsis* and rice, which indicated they may involve in drought and salinity stresses response. In addition, *MdPYL6* and *MdPYL13*, which were located in duplicated genomic regions, exhibited significant increases in expression levels following cold treatment (4 °C) (Figure 9). Meanwhile, the promoter regions of *MdPYL6* and *MdPYL13* both contained low-temperature-responsive elements (LTR) (Figure 7, Tables S3,S4). This result insinuated that these two genes will be the key candidate ABA receptors responding to cold stresses in apple. Indeed, the rice *OsPYL3* and *OsPYL9* gene, which the ortholog of *MdPYL6/13* (Figure 2 and Table S2), was reported to be involved in enhancing cold tolerance in transgenic *Arabidopsis* and rice [28,42]. 

Furthermore, many evidences have shown that PYLs involve in plant response to abiotic stress by inhibiting PP2Cs in an ABA-dependent manner [3,5,6,48]. Different works have been reported that PYLs interacted with PP2Cs in an ABA-dependent or ABA-independent manner [5,8]. For instance, AtPYL8/RCAR3 interacted with *Fagus sylvatica* FsPP2C1 in an ABA-independent manner and positively regulates ABA signaling during abiotic stress responses [48]. AtPYL5 enhanced transgenic *Arabidopsis* resistance to drought by inhibited HAB1 phosphatase activity in an ABA-dependent manner [5]. In this work, we evaluated the interactions between 13 MdPYLs and two MdPP2Cs. Our results indicated that 13 MdPYLs selectively interacted with MdPP2Cs in ABA-dependent or ABA-independent manner (Figure 10), implying the function diversity among different *MdPYLs*. In conclusion, it seems that *PYLs* have various functions in the apple and play a key role in abiotic stress induced by ABA molecular signals.

## 4. Materials and Methods 

### 4.1. Identification of MdPYL Gene Family and Chromosomal Location in Apple Genome

We used the protein sequence of 14 *AtPYLs* from the *Arabidopsis* genome to search for *MdPYLs* of the apple genome. The BLAST program was set to the default value (*e*-value < e^−10^) [49]. Manual reanalysis was performed for suspicious genes with a PYL structure and low E-value. The amino acid number, isoelectric point (pI), and molecular weight (MW) were analyzed by using the ExPASy website. The localization of *MdPYLs* on the chromosome were mapped by using the Gene Structure Display Server.

### 4.2. Phylogenetic Analysis of PYL Gene Family

The phylogenetic tree was constructed using the PYLs amino acid sequences from *M. domestica*, *A. thaliana*, *B. distachyon*, *O. sativa*, *R. communis*, *T. cacao*, *V. vinifera*, and *G. hirsutum*. Multiple alignments of protein sequences were analyzed by the neighbor-join (NJ) algorithm from 1000 repeated by MEGA 5.2 software [50].

### 4.3. Co-linear Analysis of Apple MdPYLs

The homologous genes were searched by MCScanx software [51], and the collinearity of the *MdPYLs* was obtained using the Circos program [52].

### 4.4. Gene Structure and Motif Composition of Apple MdPYLs

The MdPYLs protein sequences were found from the apple genome, and their intron–exon structures were analyzed by GSDS [53]. The MdPYL protein motifs were analyzed by Multiple EM for Motif Elicitation version 4.11.4 (MEME) [54]. The maximum number of motifs was set to 10, and the motif length was set to 6–200 amino acids.

### 4.5. Analysis of Cis-Acting Elements in the Promoter of MdPYLs

The cis-acting elements were detected using the PLACE database in the 1500 base pair (bp) upstream of the gene initiation codon (ATG) of the *MdPYLs* (Tables S3 and S4).

### 4.6. Plant Materials and Treatments

The red-fleshed apple (*Malus sieversii* f. Neidzwetzkyana (Dieck) Langenf) ‘Xinjiang No.1’, a wild apple resource with resistance to abiotic stress, was from Xinjiang of China.

The apple ‘Xinjiang No.1’ tissue culture seedlings used for stress treatment was cultured in a specific medium with Murashigeand Skoog (MS) medium, 0.8% agar, 0.5 mmol L^−1^ indole-3-butytric acid (IBA) and 0.7 mmol L^−1^ 6-benzylaminopurine (6-BA). After 30 days of growth, the ‘Xinjiang No.1’ seedlings were transferred to the medium, which supplied with 10% polyethylene glycol (PEG) 6000 or 100 mmol L^−1^ abscisic acid (ABA) or 100 mmol L^−1^ NaCl to induce different abiotic stress, respectively. Furthermore, the temperature for apple tissue culture seedlings cultured in a specific medium was adjusted to 4 °C to induce cold stress. The stress-treated seedlings were harvested at 0, 6, 12, and 24 h, then immediately frozen with liquid nitrogen and stored in a −80 °C freezer for follow-up studies.

The different apple organs, including roots, stems, leaves, pedicels, calyxes, anthers, filaments, receptacles, petals, pistils, sarcocarps, peels, young fruits (20 days after flowering), and seeds were collected from seven-year old ‘Xinjiang No.1’, which was obtained from tissue culture seedlings transplanted to the Jiao Zhou Experimental Farm (longitude 120°39′ E, latitude 36°27′ N) of Qing Dao Agricultural University.

### 4.7. Quantitative Real-Time RT-PCR Analysis

RNA was extracted from the collected samples using the EASYspin Plant RNA Rapid Extraction Kit (YPHBIO, Beijing, China). The RNA concentration was measured with a NanoDrop 2000 (Gene Company Limited, Hong Kong, China) instrument. The cDNA was obtained according to a reverse transcription kit (Takara, Dalian, China). Specific primers were designed on NCBI-BLAST (Table S5). According to the manufacturer’s instructions, quantitative real-time RT-PCR (qRT-PCR) was performed on a LightCycler^®^ 480 real-time PCR instrument (Roche, Shanghai, China). The PCR program was as followed: 95 °C for 2 min, 40 cycles of 95 °C for 30 s, 56 °C for 30 s and 72 °C for 30 s with a final dissociation stage. Actin of apple was used as an internal control for normalized gene expression levels. Repeat at least three times for each sample. Gene relative expression levels were calculated using GraphPad.Prism.5.0 software.

### 4.8. Yeast Two-Hybrid Assay

Full-length sequences of 13 MdPYLs and two MdPP2Cs (MdPP2C72: XM_008359137.2 and MdPP2C65: XM_008371984.3) were respectively amplified by PCR from ‘Xinjiang No.1’ leaf using the specific primers with enzyme digestion sites (Table S5). The full coding sequence of all MdPYLs were respectively fused to the DNA-binding domain (BD) of pGBKT7 vector, while two MdPP2Cs were respectively fused with activation domain (AD) of pGADT7 vector via double digestion technique. Combinations of pGADT7-T with pGBKT7-53 and pGBKT7-Lam were used as positive and negative controls, respectively. Combinations of empty pGADT7 with pGBKT7-MdPYLs, and empty pGBKT7 with pGADT7-MdPP2Cs were used to investigate whether MdPYLs or MdPP2Cs protein had self-activation activity. Combinations of pGBKT7-MdPYLwith pGADT7-MdPP2Cs were used to determine the potential interactions between MdPYLs and MdPP2Cs.

After co-transformation into the Y2H Gold yeast strain, the yeasts were plated on a SD/-Leu/-Trp media. Then, the positive yeasts were grown in liquid SD-Leu/Trp medium for 12 h at 30 °C. Finally, 3 μL of the co-transformed yeast cells dilutions of 10^−1^, 10^−2^, and 10^−3^ were spread on plates containing SD/-Leu/-Trp, SD/-His/-Leu/-Trp/X-α-Gal medium with 10 mM 3-AT (3-amino-1, 2, 4-triazole) and SD/-His/-Leu/-Trp/X-α-Gal medium with 10 mM 3-AT (3-amino-1, 2, 4-triazole) and 10 μM ABA. The plates were photographed after four days of incubation at 30 °C. The experiments were repeated three times.

## 5. Conclusions

Thirteen *MdPYL*s were identified in *M. domestica* genome. Phylogenetic reconstruction and gene structure analysis demonstrated that *MdPYLs* could be divided into four groups, and that they had similar gene structures and high conserved motifs in the same group. Moreover, comparative genomic analysis showed that homologs of nine *MdPYLs* were located in corresponding syntenic regions of *Arabidopsis*. The MdPYLs expression analysis in various organs revealed distinct spatiotemporal patterns. Furthermore, gene expression analysis showed that *MdPYLs* are possibly involved in multiple abiotic stress responses (ABA, salt, PEG, and cold). The results presented here call for further research aiming at revealing the potential important functions of these genes in the apple.

## Figures and Tables

**Table 1:** Basic informations of 13 *MdPYLs* identified in apple.

Gene Name	Gene ID	Genomic (bp)	CDS (bp)	Exon	pI	MW (kDa)	Protein (aa)
*MdPYL1*	MD01G1078900	3252	555	3	6.45	20.80	184
*MdPYL2*	MD01G1158500	624	624	1	6.62	22.75	207
*MdPYL3*	MD01G1216100	3049	597	3	5.97	21.80	198
*MdPYL4*	MD04G1165000	1680	777	2	6.13	27.66	258
*MdPYL5*	MD05G1300200	558	558	1	5.82	20.13	185
*MdPYL6*	MD06G1034000	621	621	1	5.37	22.90	206
*MdPYL7*	MD07G1147700	2963	555	3	6.30	20.77	184
*MdPYL8*	MD07G1227100	624	624	1	6.44	22.74	207
*MdPYL9*	MD07G1286000	2576	615	3	6.06	22.33	204
*MdPYL10*	MD08G1043500	606	606	1	5.02	22.12	201
*MdPYL11*	MD12G1178800	2150	732	2	8.30	26.16	243
*MdPYL12*	MD15G1060800	609	609	1	5.20	22.04	202
*MdPYL13*	MD16G1274400	621	621	1	5.37	23.14	206

## Supplementary Materials

Supplementary materials can be found at https://www.mdpi.com/2223-7747/9/8/1028/s1. Table S1: Amino acid sequences of the plant *PYLs*. Table S2: The clustered groups of PYL proteins from eight species. Table S3: The 1500 bp promoter sequences of the 13 *MdPYL*s. Table S4: Cis-element analysisof the *MdPYL*s promoter regions in the apple. Table S5: Primers used in this study. Figure S1: The results of agarose gel electrophoresis test of 13 MdPYLs and two MdPP2Cs. Figure S2: Self activation analysis of 13 MdPYLs and two MdPP2Cs fusions.

## References

[1] Cutler S.R., Rodriguez P.L., Finkelstein R.R., Abrams S.R. (2010). Abscisic acid: Emergence of a core signaling network. Annu. Rev. Plant. Biol..

[2] Lee S.C., Luan S. (2012). ABA signal transduction at the crossroad of biotic and abiotic stress responses. Plant. Cell Environ..

[3] Fujii H., Chinnusamy V., Rodrigues A., Rubio S., Antoni R., Park S.Y., Cutler S.R., Sheen J., Rodriguez P.L., Zhu J.K. (2009). In vitro reconstitution of an abscisic acid signalling pathway. Nature.

[4] Park S.Y., Fung P., Nishimura N., Jensen D.R., Fujii H., Zhao Y., Lumba S., Santiago J., Rodrigues A., Chow T.F. (2009). Abscisic acid inhibits type 2C protein phosphatases via the PYR/PYL family of START proteins. Science.

[5] Santiago J., Rodrigues A., Saez A., Rubio S., Antoni R., Dupeux F., Park S.Y., Márquez J.A., Cutler S.R., Rodriguez P.L. (2009). Modulation of drought resistance by the abscisic acid receptor PYL5 through inhibition of clade A PP2Cs. Plant. J..

[6] Ma Y., Szostkiewicz I., Korte A., Moes D., Yang Y., Christmann A., Grill E. (2009). Regulators of PP2C phosphatase activity function as abscisic acid sensors. Science.

[7] Miyazono K., Miyakawa T., Sawano Y., Kubota K., Kang H.J., Asano A., Miyauchi Y., Takahashi M., Zhi Y., Fujita Y. (2009). Structural basis of abscisic acid signalling. Nature.

[8] Nishimura N., Hitomi K., Arvai A.S., Rambo R.P., Hitomi C., Cutler S.R., Schroeder J.I., Getzoff E.D. (2009). Structural mechanism of abscisic acid binding and signaling by dimeric PYR1. Science.

[9] Peterson F.C., Burgie E.S., Park S.Y., Jensen D.R., Weiner J.J., Bingman C.A., Chang C.E., Cutler S.R., Phillips G.N., Volkman B.F. (2010). Structural basis for selective activation of ABA receptors. Nat. Struct. Mol. Biol..

[10] Miyakawa T., Fujita Y., Yamaguchi-Shinozaki K., Tanokura M. (2013). Structure and function of abscisic acid receptors. Trends. Plant. Sci..

[11] Santiago J., Dupeux F., Betz K., Antoni R., Gonzalez-Guzman M., Rodriguez L., Márquez J.A., Rodriguez P.L. (2012). Structural insights into PYR/PYL/RCAR ABA receptors and PP2Cs. Plant. Sci..

[12] Hao Q., Yin P., Li W., Wang L., Yan C., Lin Z., Wu J.Z., Wang J., Yan S.F., Yan N. (2011). The molecular basis of ABA-independent inhibition of PP2Cs by a subclass of PYL proteins. Mol. Cell..

[13] Li W., Wang L., Sheng X., Yan C., Zhou R., Hang J., Yin P., Yan N. (2013). Molecular basis for the selective and ABA-independent inhibition of PP2CA by PYL13. Cell Res..

[14] Gonzalez-Guzman M., Pizzio G.A., Antoni R., Vera-Sirera F., Merilo E., Bassel G.W., Fernandez M.A., Holdsworth M.J., Perez-Amador M.A., Kollist H. (2012). Arabidopsis PYR/PYL/RCAR receptors play a major role in quantitative regulation of stomatal aperture and transcriptional response to abscisic acid. Plant. Cell.

[15] Zhao Y., Chan Z., Gao J., Xing L., Cao M., Yu C., Hu Y., You J., Shi H., Zhu Y. (2016). ABA receptor PYL9 promotes drought resistance and leaf senescence. Proc. Natl. Acad. Sci. USA.

[16] Antoni R., Gonzalez-Guzman M., Rodriguez L., Peirats-Llobet M., Pizzio G.A., Fernandez M.A., De Winne N., De Jaeger G., Dietrich D., Bennett M.J. (2013). PYRABACTIN RESISTANCE1-LIKE8 plays an important role for the regulation of abscisic acid signaling in root. Plant. Physiol..

[17] Xing L., Zhao Y., Gao J., Xiang C., Zhu J.K. (2016). The ABA receptor PYL9 together with PYL8 plays an important role in regulating lateral root growth. Sci. Rep..

[18] Zhao Y., Xing L., Wang X., Hou Y.J., Gao J., Wang P., Duan C.G., Zhu X., Zhu J.K. (2014). The ABA receptor PYL8 promotes lateral root growth by enhancing MYB77-dependent transcription of auxin-responsive genes. Sci. Signal..

[19] Romero P., Lafuente M.T., Rodrigo M.J. (2012). The Citrus ABA signalosome: Identification and transcriptional regulation during sweet orange fruit ripening and leaf dehydration. J. Exp. Bot..

[20] Boneh U., Biton I., Zheng C., Schwartz A., Ben-Ari G. (2012). Characterization of potential ABA receptors in Vitis vinifera. Plant. Cell Rep..

[21] He Y., Hao Q., Li W., Yan C., Yan N., Yin P. (2014). Identification and characterization of ABA receptors in Oryza sativa. PLoS ONE.

[22] González-Guzmán M., Rodríguez L., Lorenzo-Orts L., Pons C., Sarrión-Perdigones A., Fernández M.A., Peirats-Llobet M., Forment J., Moreno-Alvero M., Cutler S.R. (2014). Tomato PYR/PYL/RCAR abscisic acid receptors show high expression in root, differential sensitivity to the abscisic acid agonist quinabactin, and the capability to enhance plant drought resistance. J. Exp. Bot..

[23] Guo D., Zhou Y., Li H.L., Zhu J.H., Wang Y., Chen X.T., Peng S.Q. (2017). Identification and characterization of the abscisic acid (ABA) receptor gene family and its expression in response to hormones in the rubber tree. Sci. Rep..

[24] Li Y., Wang D., Sun C., Hu X., Mu X., Hu J., Yang Y., Zhang Y., Xie C.G., Zhou X. (2017). Molecular characterization of an AtPYL1-like protein, BrPYL1, as a putative ABA receptor in Brassica rapa. Biochem. Biophys. Res. Commun..

[25] Zhang G., Lu T., Miao W., Sun L., Tian M., Wang J., Hao F. (2017). Genome-wide identification of ABA receptor PYL family and expression analysis of PYLs in response to ABA and osmotic stress in Gossypium. Peer J..

[26] Bai G., Xie H., Yao H., Li F., Chen X., Zhang Y., Xiao B., Yang J., Li Y., Yang D.H. (2019). Genome-wide identification and characterization of ABA receptor PYL/RCAR gene family reveals evolution and roles in drought stress in Nicotiana tabacum. BMC Genom..

[27] Chen Y., Feng L., Wei N., Liu Z.H., Hu S., Li X.B. (2017). Overexpression of cotton PYL genes in Arabidopsis enhances the transgenic plant tolerance to drought stress. Plant. Physiol. Biochem..

[28] Lenka S.K., Muthusamy S.K., Chinnusamy V., Bansal K.C. (2018). Ectopic Expression of Rice PYL3 Enhances Cold and Drought Tolerance in Arabidopsis thaliana. Mol. Biotechnol..

[29] He Z., Zhong J., Sun X., Wang B., Terzaghi W., Dai M. (2018). The Maize ABA Receptors ZmPYL8, 9, and 12 Facilitate Plant Drought Resistance. Front. Plant. Sci..

[30] Kim H., Hwang H., Hong J.W., Lee Y.N., Ahn I.P., Yoon I.S., Yoo S.D., Lee S., Lee S.C., Kim B.G. (2012). A rice orthologue of the ABA receptor, OsPYL/RCAR5, is a positive regulator of the ABA signal transduction pathway in seed germination and early seedling growth. J. Exp. Bot..

[31] Kim H., Lee K., Hwang H., Bhatnagar N., Kim D.Y., Yoon I.S., Byun M.O., Kim S.T., Jung K.H., Kim B.G. (2014). Overexpression of PYL5 in rice enhances drought tolerance, inhibits growth, and modulates gene expression. J. Exp. Bot..

[32] Miao C., Xiao L., Hua K., Zou C., Zhao Y., Bressan R.A., Zhu J.K. (2018). Mutations in a subfamily of abscisic acid receptor genes promote rice growth and productivity. Proc. Natl. Acad. Sci. USA.

[33] Pizzio G.A., Rodriguez L., Antoni R., Gonzalez-Guzman M., Yunta C., Merilo E., Kollist H., Albert A., Rodriguez P.L. (2013). The PYL4 A194T mutant uncovers a key role of PYR1-LIKE4/PROTEIN PHOSPHATASE 2CA interaction for abscisic acid signaling and plant drought resistance. Plant. Physiol..

[34] Liang C., Liu Y., Li Y., Meng Z., Yan R., Zhu T., Wang Y., Kang S., Ali Abid M., Malik W. (2017). Activation of ABA Receptors Gene GhPYL9-11A Is Positively Correlated with Cotton Drought Tolerance in Transgenic Arabidopsis. Front. Plant. Sci..

[35] Yu J., Yang L., Liu X., Tang R., Wang Y., Ge H., Wu M., Zhang J., Zhao F., Luan S. (2016). Overexpression of Poplar Pyrabactin Resistance-Like Abscisic Acid Receptors Promotes Abscisic Acid Sensitivity and Drought Resistance in Transgenic Arabidopsis. PLoS ONE.

[36] Li G., Xin H., Zheng X.F., Li S., Hu Z. (2012). Identification of the abscisic acid receptor VvPYL1 in Vitis vinifera. Plant. Biol. (Stuttg).

[37] Boneh U., Biton I., Schwartz A., Ben-Ari G. (2012). Characterization of the ABA signal transduction pathway in Vitis vinifera. Plant. Sci..

[38] Yin P., Fan H., Hao Q., Yuan X., Wu D., Pang Y., Yan C., Li W., Wang J., Yan N. (2009). Structural insights into the mechanism of abscisic acid signaling by PYL proteins. Nat. Struct. Mol. Biol..

[39] Fuchs S., Tischer S.V., Wunschel C., Christmann A., Grill E. (2014). Abscisic acid sensor RCAR7/PYL13, specific regulator of protein phosphatase coreceptors. Proc. Natl. Acad. Sci. USA.

[40] Bai G., Yang D.H., Zhao Y., Ha S., Yang F., Ma J., Gao X.S., Wang Z.M., Zhu J.K. (2013). Interactions between soybean ABA receptors and type 2C protein phosphatases. Plant. Mol. Biol..

[41] Di F., Jian H., Wang T., Chen X., Ding Y., Du H., Lu K., Li J., Liu L. (2018). Genome-Wide Analysis of the PYL Gene Family and Identification of PYL Genes That Respond to Abiotic Stress in Brassica napus. Genes.

[42] Tian X., Wang Z., Li X., Lv T., Liu H., Wang L., Niu H., Bu Q. (2015). Characterization and Functional Analysis of Pyrabactin Resistance-Like Abscisic Acid Receptor Family in Rice. Rice.

[43] Fan W., Zhao M., Li S., Bai X., Li J., Meng H., Mu Z. (2016). Contrasting transcriptional responses of PYR1/PYL/RCAR ABA receptors to ABA or dehydration stress between maize seedling leaves and roots. BMC Plant. Biol..

[44] Liu J., Zhao F.L., Guo Y., Fan X.C., Wang Y.j., Wen Y.Q. (2019). The ABA receptor-like gene VyPYL9 from drought-resistance wild grapevine confers drought tolerance and ABA hypersensitivity in Arabidopsis. Plant. Cell Tissue Organ. Cult..

[45] Li X., Li G., Li Y., Kong X., Zhang L., Wang J., Li X., Yang Y. (2018). ABA Receptor Subfamily III Enhances Abscisic Acid Sensitivity and Improves the Drought Tolerance of Arabidopsis. Int. J. Mol. Sci..

[46] Chen Z., Kong L., Zhou Y., Chen Z., Tian D., Lin Y., Wang F., Chen S. (2017). Endosperm-specific OsPYL8 and OsPYL9 act as positive regulators of the ABA signaling pathway in rice seed germination. Funct. Plant. Biol..

[47] Han Y.L., Jang G., Um T., Kim J.K., Lee J.S., Yang D.C. (2015). The soluble ABA receptor PYL8 regulates drought resistance by controlling ABA signaling in Arabidopsis. Plant. Biotechnol. Rep..

[48] Saavedra X., Modrego A., Rodríguez D., González-García M.P., Sanz L., Nicolás G., Lorenzo O. (2010). The nuclear interactor PYL8/RCAR3 of Fagus sylvatica FsPP2C1 is a positive regulator of abscisic acid signaling in seeds and stress. Plant. Physiol..

[49] Altschul S.F., Madden T.L., Schaffer A.A., Zhang J., Zhang Z., Miller W., Lipman D.J. (1997). Gapped BLAST and PSI-BLAST: A new generation of protein database search programs. Nucleic Acids Res..

[50] Felsenstein J. (1985). Confidence Limits on Phylogenies: An Approach Using the Bootstrap. Evolution.

[51] Wang Y., Tang H., Debarry J.D., Tan X., Li J., Wang X., Lee T.H., Jin H., Marler B., Guo H. (2012). MCScanX: A toolkit for detection and evolutionary analysis of gene synteny and collinearity. Nucleic Acids Res..

[52] Krzywinski M., Schein J., Birol I., Connors J., Gascoyne R., Horsman D., Jones S.J., Marra M.A. (2009). Circos: An information aesthetic for comparative genomics. Genome Res..

[53] Hu B., Jin J., Guo A.Y., Zhang H., Luo J., Gao G. (2015). GSDS 2.0: An upgraded gene feature visualization server. Bioinformatics.

[54] Bailey T.L., Boden M., Buske F.A., Frith M., Grant C.E., Clementi L., Ren J., Li W.W., Noble W.S. (2009). MEME SUITE: Tools for motif discovery and searching. Nucleic Acids Res..

